# Characterization and Genomic Analysis of a Highly Efficient Dibutyl Phthalate-Degrading Bacterium *Gordonia* sp. Strain QH-12

**DOI:** 10.3390/ijms17071012

**Published:** 2016-06-25

**Authors:** Decai Jin, Xiao Kong, Huijun Liu, Xinxin Wang, Ye Deng, Minghong Jia, Xiangyang Yu

**Affiliations:** 1 CAS Key Laboratory of Environmental Biotechnology, Research Center for Eco-Environmental Sciences, Chinese Academy of Sciences, Beijing 100085, China; dcjin@rcees.ac.cn (D.J.); kongxiaozaikeda@163.com (X.K.); 2 Beijing Key Laboratory of Detection and Control of Spoilage Organisms and Pesticide Residues in Agricultural Products, Beijing University of Agriculture, Beijing 102206, China; huijunliu78@163.com; 3 China Offshore Environmental Service Co., Ltd., Tianjin 300452, China; wangxx200899@163.com; 4 Institute of Food Quality and Safety, Jiangsu Academy of Agricultural Sciences, Nanjing 210014, China; yuxy@jaas.ac.cn

**Keywords:** phthalate esters, phthalic acid, *Gordonia* sp., biodegradation, genome sequencing

## Abstract

A bacterial strain QH-12 isolated from activated sludge was identified as *Gordonia* sp. based on analysis of 16S rRNA gene sequence and was found to be capable of utilizing dibutyl phthalate (DBP) and other common phthalate esters (PAEs) as the sole carbon and energy source. The degradation kinetics of DBP under different concentrations by the strain QH-12 fit well with the modified Gompertz model (*R*^2^ > 0.98). However, strain QH-12 could not utilize the major intermediate product phthalate (phthalic acid; PA) as the sole carbon and energy source, and only a little amount of PA was detected. The QH-12 genome analysis revealed the presence of putative hydrolase/esterase genes involved in PAEs-degradation but no phthalic acid catabolic gene cluster was found, suggesting that a novel degradation pathway of PAEs was present in *Gordonia* sp. QH-12. This information will be valuable for obtaining a more holistic understanding on diverse genetic mechanisms of PAEs-degrading *Gordonia* sp. strains.

## 1. Introduction

The occurrence of phthalate esters (PAEs) in soil and water is a major threat to human health. PAEs have been frequently detected from the disposal of manufacturing, producing, and leaching from plastics, processing and industrial wastes, municipal solid waste, and other phthalates-containing products [1]. Dibutyl phthalate (DBP), one of the most widely used phthalate esters with the increasing market demand for plasticizers, was frequently detected in water, plants, soil, sediment, air, food, and even in human breast milk, blood or serum, and urine [2,3,4]. Nowadays, DBP has been shown to disrupt the endocrine systems of humans and wildlife [5,6] and thus is identified as a priority controlled hazardous substance by the United States Environmental Protection Agency (US EPA) and China National Environmental Monitoring Center [7].

The environmental fate of PAEs has caused great concern and microbial degradation is considered one of the major routes under aerobic and anaerobic conditions although hydrolysis, photolysis and volatilization of PAEs can occur [8]. During the past few decades, many bacteria strains with the ability to utilize PAEs and their isomers as sole carbon and energy sources have been isolated and characterized, such as *Acinetobacter* sp. M673 [9], *Micrococcus* sp. YGJ1 [10], *Gordonia* sp. QH-11 [11], *Rhodococcus jostii* RHA1 [12], *Variovorax* sp. BS1 [13], *Pseudoxanthomonas* sp. [8], and *Sphingobium* sp. SM42 [14]. Most of these reported strains could only transform PAEs to their corresponding monoesters of phthalate or phthalic acid. In most habitats, at least two types of strains are required for the complete degradation of PAEs. Among the PAEs-degrading bacteria strains, actinomycetes play significant roles in PAEs removal [11,15,16,17,18,19], especially for genus *Gordonia*. So far, ten PAEs-degrading strains belonged to the genus *Gordonia* have been isolated from various environmental metrics. Of these *Gordonia* strains, *Gordonia* sp. strain MTCC 4818 can use BBP as sole carbon and energy sources, which resulted in the accumulation of monobutyl phthalate (MBuP) and monobenzyl phthalate (MBzP) in the culture medium [15]; *Gordonia* sp. strain JDC-2 rapidly degraded di-n-octyl phthalate (DOP) into phthalic acid (PA) without further degradation [16]; *Gordonia* sp. JDC2, JDC13, JDC26, and JDC33 found to utilize DBP as the sole source of carbon and energy but unable to utilize PA, though 3,4-phthalate dioxygenase gene has been successfully cloned [17]; *Gordonia* sp. QH-11 could utilize PAEs and the main intermediate product PA [11]; DnOP can be completely degraded by a single bacterial strain, *Gordonia* sp. strain Dop5 [18]; *Gordonia* sp. strain HS-NH1 which was isolated from activated sludge in south lake (Wuhan), has the capacity to utilize various PAEs and major intermediate products were PA and protocatechuate acid (PCA) [19]. Nevertheless, very little information was available on metabolically diverse mechanism of PAEs by genus *Gordonia*. Thus, it is of great importance to isolate and characterize more *Gordonia* strains for PAEs degradation and improve our knowledge of the PAEs degradation mechanism by genus *Gordonia*. In addition, research focused on the pathway elucidation of PAEs degradation in *Gordonia* strains based on genome analysis is still in its infancy stage. So far, only one draft genome of PAEs-degrading *Gordonia* strains has been published recently [19]. Further, investigation of the PAEs degradation mechanism by the genus *Gordonia* based on genomics is still important because it is one of the most frequently reported bacterial strains for PAEs degradation.

In this study, we described the isolation and identification of a DBP-degrading *Gordonia* sp. strain QH-12, which could not utilize PA to grow. Kinetics of DBP degradation by strain QH-12 was also investigated. In addition, we employed whole genome shotgun sequencing to putatively assign genes expected to be involved in PAEs metabolism, and also proposed the reason for why *Gordonia* sp. strain QH-12 was unable to degrade PA based on genome sequencing analysis.

## 2. Results and Discussion

### 2.1. Isolation and Identification of PAEs-Degrading Strains

**Figure 1:**
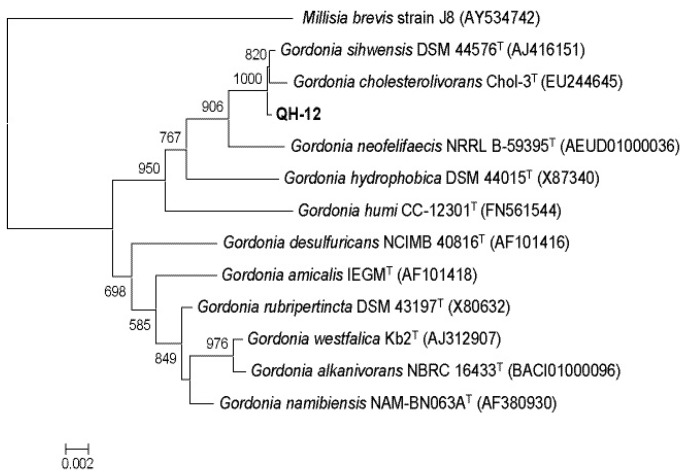
Phylogenetic tree derived from 16S rRNA gene sequence of strain QH-12 and sequences of related species. Distances were calculated using neighbor-joining method. Numbers at branch points are bootstrap values (based on 1000 samplings). *Millisia brevis* strain J81 (AY534742) was used as the out-group.

Several bacterial strains were isolated from activated sludge after enriched in mixed PAEs-containing medium for approximately two months. One pure gram-positive bacteria strain, named QH-12 was selected for further study. QH-12 was aerobic, non-motile, and short-rod shaped. The phylogenetic relationships based on the 16S rRNA gene sequences were closest to that *Gordonia cholesterolivorans* Chol-3^T^ (EU244645) (>99.0% similarity). Based on the results of morphological characteristics and 16S rRNA gene sequence analysis, strain QH-12 was identified as a novel *Gordonia* sp. strain (Figure 1).

Recently, several *Gordonia* stains have been reported to be able to degrade various PAEs [11,15,16,17,18]. In our previous study, *Gordonia* sp. QH-11 was isolated from the same sites and has the capacity to utilize DBP and other PAEs, strain QH-11 shows most similarities to *Gordonia hydrophobica* based on its 16S rRNA gene sequence. Interestingly, in this study a different *Gordonia* strain was present in the same environment niche and both of them can degrade PAEs. Therefore, this result implies that genus *Gordonia* may have great potential in PAEs removal in sewage treatment plants using A2/O process.

### 2.2. Biodegradation Kinetics of DBP by Gordonia sp. Strain QH-12

**Figure 2:**
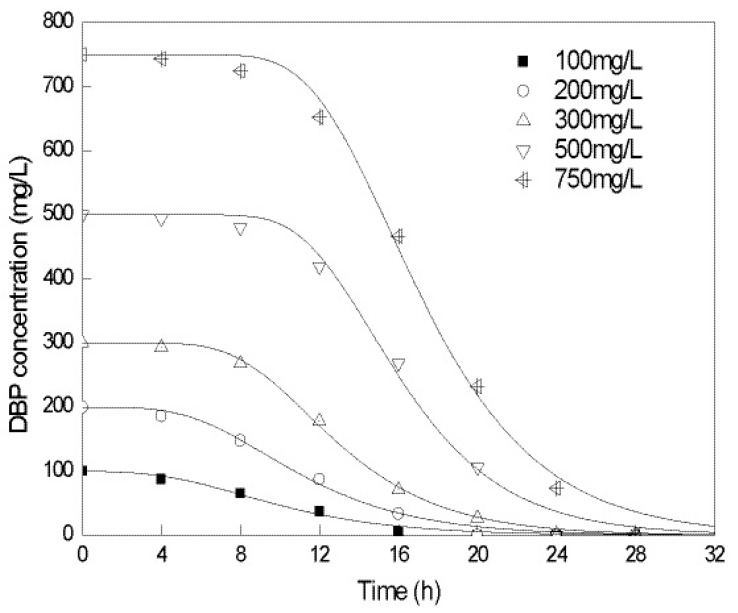
Best-fitted curves for DBP degradation using modified Gompertz mode at different initial concentrations by *Gordonia* sp. QH-12

The batch experiments showed that the optimal pH and temperature for DBP degradation by strain QH-12 were 7.0 and 30 °C, respectively (data not shown). Under optimal growth conditions, the degradation curves of varying initial concentrations (100−750 mg/L) of DBP by whole cells of *Gordonia* sp. QH-12 were compared in Figure 2. The results showed that the degradation of DBP followed the modified Gompertz model, which could be expressed as

(1) $$S=S_{0}\left\{1-\text{exp}\left\{-\text{exp}\left[\frac{eR_{m}}{S_{0}}\left(\lambda -t\right)+1\right]\right\}\right\}$$

where *S* is the time-course concentration of the tested substrate (mg/L), *S*_0_ is the initial concentration of the tested substrate (mg/L), *R*m is the maximum transformation rate, *λ* is the lag phase time (h), and *t* is the incubation time (h).

As shown in Figure 2, all the DBP was completely degraded by strain QH-12 within 32 h of incubation, and the DBP depletion curves are described well by the modified Gompertz model with a high correlation coefficient (*R*^2^ > 0.98) (Table 1). These results suggest that the lag phase was extended as the concentrations of DBP increased, which may be attributed to the higher toxicity of DBP in higher concentrations. For example, when the concentration of DBP increased from 100 to 750 mg/L, the lag phase value increased from 3.83 to 11.43 h, respectively. A similar trend was observed for the maximum transformation rate (*R*_m_), which increased from 8.67 to 66.36 mg/L/h. Recently, the modified Gompertz model has frequently been used to describe the degradation kinetics of PAEs. In our previous study, the degradation of DBP at concentrations of 100−750 mg/L by *Gordonia* sp. QH-11 isolated from the same sites also followed the modified Gompertz model and 500 mg/L could be completely degraded within 45 h [11]. In 2007, degradation curve of DEP at moderate concentrations (39−610 mg/L) by a strain of *Sphingomonas* was successfully fitted by modified Gompertz model [20]. In addition, Wang et al. [21] also used this formula to analyze dimethyl phthalate (200−800 mg/L) transformation kinetics. However, the first-order kinetics was the most widely used model for describing the biodegradation of DBP. For instance, Wu et al. [22] found that biodegradation of DBP by *Agrobacterium* sp. JDC-49 fitted well with first-order kinetics and strain JDC-49 could completely degrade DBP within 48 h when the initial concentration was lower than 200 mg/L. In addition, Fang et al. [23] reported DBP biodegradation by *Enterobacter* sp. T5 fitted a first-order kinetic model when the initial concentrations of DBP were <1000 mg/L and the degradation half-life was about 21.0 h when DBP concentration was 500 mg/L. Moreover, Xu et al. [24] reported that DBP biodegradation by *Pseudomonas fluorescens* B-1 conformed to the first-order kinetic model at initial concentrations of 2.5–10 mg/L and the half-life value was increased from approximately 14 to 24 h, respectively. Thus, comparative evaluation indicates that DBP biodegradation efficiency of *Gordonia* sp. QH-12 was relatively higher although marginal inhibition at higher substrate concentrations was noted.

### 2.3. Substrate Spectrum of Gordonia sp. Strain QH-12

The degradation of individual PAEs (DMP, DEP, DBP, DOP, DIOP, and DEHP) and most commonly detected intermediates of PAEs (MBP, phthalic acid, and protocatechuic acid) were measured (Table 2).

**Figure 3:**
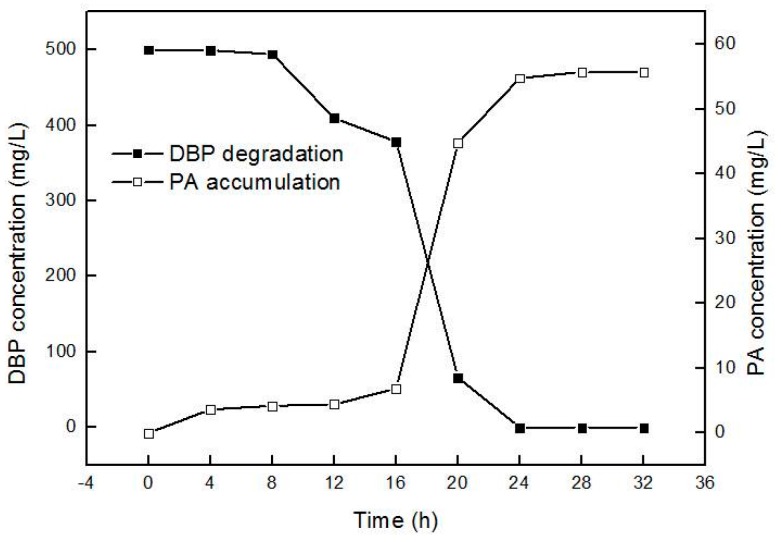
PA accumulation during DBP degradation by QH-12.

Despite its ability to transform all dialkyl PAEs tested and MBP which was initial intermediate product of DBP, strain QH-12 failed to grow on phthalic acid and protocatechuic acid under our experimental conditions. As shown in Figure 3, the concentration of PA, the initial intermediate of DBP, increased, whereas the relative fraction of DBP parent decreased, indicating a progressive conversion of DBP to metabolites by strain QH-12. For example, 500 mg/L of DBP was degraded completely within 24 h, and the concentration of PA reached 55.73 mg/L in the liquid medium and almost no change occurred after approximately a month (data not shown). As only 18.90 mol % of PA was formed, this result implied that PA was a main dead-end product of DBP biodegradation by *Gordonia* sp. QH-12. We have tried hardest to detect the other degradation products of DBP by strain QH-12 (GC-MS analysis). However, besides DBP and PA, only the phthalic anhydride was also detected (a small peak), which is inconsistent with the results reported by many previous studies [15,16,17]. In addition, Jin et al. [11] reported a first *Gordonia* strain which can degrade PAEs and its metabolite PA simultaneously and the gene encoding a large subunit of phthalate 3,4-dioxygenase was present in the strain QH-11; Thus, further analysis on *Gordonia* sp. QH-12 genomics is necessary to explain why it cannot further transform PA.

### 2.4. Overview of the Gordonia sp. Strain QH-12 Genome

**Figure 4:**
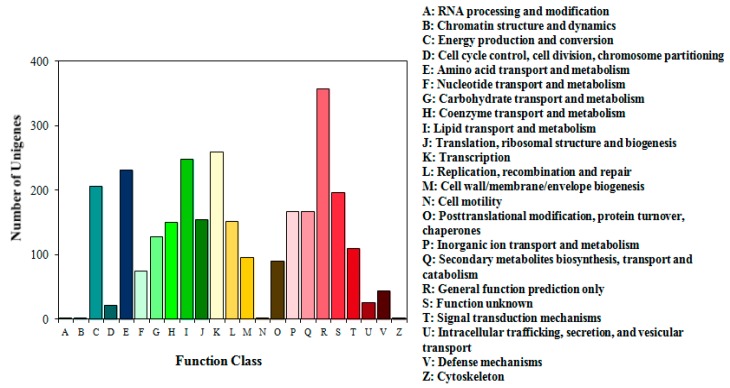
COG function classification of strain QH-12.

The draft genome of strain QH-12 has a single chromosome of 3,901,461 bp with a G + C content of 68.4%. The genome contains 3429 predicted protein coding genes (CDS) with an average size of 984 bp, giving a coding intensity of 86.50%. Analysis revealed 33 pseudogenes, 42 tRNA genes, 1 noncoding RNA (ncRNA), and 4 rRNA operons in the genome. Average nucleotide identity (ANI) analysis revealed that *Gordonia* sp. QH-12 is phylogenetically related to *Gordonia sihwensis* NBRC 108236 (99.18%), *Gordonia* sp. no. 9 (99.13%), and *Gordonia neofelifaecis* NRRL B-59395 (81.97%). Of the 3,429 CDS, 2579 could be assigned to 23 different categories of clusters of orthologous groups (COGs). These results clearly suggest the organism’s efficient lipid, carbohydrate, and amino acid transport and metabolism for energy (Figure 4).

In the present study, *Gordonia* sp. strain QH-12 was found to be able to convert phthalate esters to phthalic acid. According to sequence analysis, there were 16 and 36 genes encoding for putative esterase/carboxylesterase and hydrolase/alpha/beta hydrolase, respectively. Among these esterases or hydrolases, several gene-encoding enzymes showed relatively low amino acid similarities to those of previously reported enzymes for phthalate esters decomposed. For example, Y710_03810 from QH-12 showed 26% identity with monoethylhexylphthalate hydrolase (accession no. AB214635) from *Gordonia* sp. P8219 with 90% coverage [25]; Y710_01560 from QH-12 showed 26% identity with carboxyl esterase (accession no. KM098150) from *B. subtilis* K91 with 96% coverage [26]. It is worth noting that amino acid sequence of alpha/beta hydrolase (Y710_03635) showed 42%, 38%, 33%, and 45% identity to esterase (accession no. AEW03609) from *Sulfobacillus acidophilus* DSM10332 [27], a cold-active phthalate esters hydrolase (accession no. KC438416) from screening of a metagenomic library [28], DBP hydrolase (accession no. JQ478494) from *Acinetobacter* sp. M673 [9], and esterase (accession no. JQ478494) from *Sphingobium* sp. SM42 [14], respectively at high coverage. Cloning and expression of these putative esterases and hydrolases would be helpful for better understanding the mechanism of PAEs-degradation in genus *Gordonia*.

### 2.5. Elucidating the Molecular Mechanism for Inability to Degrade PA in Gordonia Strains

It is well known that phthalate could be converted to protocatechuate by phthalate catabolic gene cluster (*pht*). In general, the whole *pht* cluster consisted of seven functional genes (*phtAaAbAcAdBCR*) and an unknown ORF (*phtU*). Of the seven functional genes in Gram-positive bacteria, *phtAaAbAcAd* genes encode dioxygenase which can oxygenate phthalate to 3, 4-dihydro-3,4-dihydroxyphthalate, and then 3,4-dihydro-3,4-dihydroxyphthalate was dehydrogenated to 3,4-dihydroxyphthalate by dehydrogenase (*phtB*) and finally 3,4-dihydroxyphthalate was decarboxylated to form protocatechuate by decarboxylase (*phtC*). In this study, no phthalate degradation was observed, and it was not surprising that no phthalate catabolic gene cluster was identified via genomic analysis of strain QH-12. In 2015, a complete genome of *Gordonia* sp. strain QH-11 which can utilize phthalate was available in GenBank (CP011853) and the whole *pht* gene can be found in chromosome of this strain. Recently, the draft genome of another PAEs and phthalate-utilizing *Gordonia* sp. strain HS-NH1 has been sequenced and the *pht* operon (*phtBAaAbUAcAdCR*) was annotated and characterized [19]. Besides strains *Gordonia* sp. QH-11 and HS-NH1, comparative genome analysis of *Gordonia* spp. found that only two draft genome of *Gordonia* strains harbor *pht* gene cluster. However, the incomplete *pht* gene cluster was observed in both of these two *Gordonia* strains (*Gordonia sihwensis* NBRC 108236 and *Gordonia namibiensis* NBRC 108229). For example, *phtAaAbUAcAdC* and *phtAaAbUBAcAd* were found in draft genome of *Gordonia sihwensis* NBRC 108236 and *Gordonia namibiensis* NBRC 108229, respectively. As shown in Figure 1, the 16S rRNA gene sequence of strain QH-12 showed a highest similarity to that of *Gordonia sihwensis* NBRC 108236. In the past years, most of *Gordonia* strains were reported to be unable to utilize phthalate. The reason for this phenomenon may be attributed to gene lacking or incomplete *pht* gene clusters, suggesting that a novel degradation pathway of PAEs, particularly for MBP metabolites, was present in strain QH-12. Construction of genetically engineered bacteria can overcome the incomplete degradation of phthalate esters during bioremediation process. Further experiments have to be carried out to elucidate this possibility.

## 3. Materials and Methods

### 3.1. Chemicals and Media

Dimethyl phthalate (DMP), diethyl phthalate (DEP), Di-n-butyl phthalate (DBP), di (2-ethylheyl) phthalate (DEHP), di-n-octyl phthalate (DOP), and phenol were purchased from Alfa Aesar (Ward Hill, MA, USA). The purity of these chemicals is greater than 98%. Methanol and ethyl acetates (high-performance liquid chromatography grade) were purchased from Fisher Scientific (Shanghai, China). All other chemicals and solvents were of analytical grade. Luria-Bertani (LB) liquid medium (adjusted to pH 7.2 with NaOH) consisted of (per liter of distilled water) 10 g of tryptone, 5.0 g of yeast extract, 5.0 g of sodium chloride. Mineral salt medium (MSM) contained the following components (L^−1^): 5.8 g of K_2_HPO_4_, 4.5 g of KH_2_PO_4_, 2.0 g of (NH_4_)_2_SO_4_, 0.16 g of MgCl_2_, 0.02 g of CaCl_2_, 0.0024 g of Na_2_MoO_4_·2H_2_O, 0.0018 g of FeCl_3_, and 0.0015 g of MnCl_2_·2H_2_O. The pH was adjusted to pH 7.0. The concentration of PAEs added to the MSM was based on the requirement of each experiment. All solid medium contained 2.0% agar.

### 3.2. Isolation and Identification of DBP-Degrading Microorganism

The activated sludge sample was obtained from an aerobic tank of a wastewater treatment plant with the A2/O process located in a northern suburb of Beijing, China. To enrich the PAEs-degrading microorganisms, the sample was cultured in 250 mL bottles with 100 mL MSM at 30 °C and 150 rpm for 7–10 days. The procedure of enrichment and isolation was conducted as described by Jin et al. [29]. Pure cultures were phylogenetically characterized using 16S rRNA gene sequencing. Universal primers 27f/1492r [30] was used for PCR amplification of the 16S rRNA genes. The PCR thermal cycling consisted of an initial denaturation at 95 °C for 10 min, followed by 35 cycles at 94 °C for 45 s, 56 °C for 45 s, and 72 °C for 90 s, plus a final step at 72 °C for 10 min. The obtained PCR product was cloned and further sequenced. The obtained sequences were subjected to BLAST homology search (http://www.ncbi.nlm.nih.gov/BLAST).

### 3.3. Biodegradation Kinetic of DBP by Gordonia sp. Strain QH-12

To determine the microbial degradation of PAEs by the QH-12 strain, batch experiments were performed as described by [11]. Briefly, five initial concentrations (100 mg/L, 200 mg/L, 300 mg/L, 500 mg/L, and 750 mg/L) of DBP were used, 1 mL bacterial inoculum (OD_600_ = 0.2) was added to 150 mL Erlenmeyer flasks containing 50 mL sterilized MSM supplemented with various concentrations of DBP. The flask was placed on a 150 rpm shaker maintained at 30 °C and pH 7.0. Samples were collected every 4 h during a period of 32 h, and HPLC analysis was performed to detect the DBP residue. Each experiment was performed in triplicate.

### 3.4. Substrate Utilization Experiments

To examine the ability of strain QH-12 to degrade different substrates, batch experiments of QH-12 were conducted in 250 mL bottles with 100 mL MSM, and the initial concentration of each substrate was 200 mg/L. Several important compounds were selected, including DMP, DEP, DBP, DOP, DIOP, DEHP, mono-n-butyl phthalate (MBP), PA, and protocatechuic acid (PCA). The initial concentration of each substrate was 200 mg/L, and substrate utilization was assessed by microbial growth through measuring the increase of the biomass (OD_600_) combined with the visible turbidity after 36 h of incubation.

### 3.5. Genome Sequencing, Assembly, and Annotation

High-molecular-mass genomic DNA was isolated from *Gordonia* sp. strain QH-12 using a commercial DNA isolation kit (Omega Bio-tek, Inc., Norcross, GA, USA) and then sequenced using an Illumina HiSeq 2000 platform (San Diego, CA, USA) with a whole-genome shotgun (WGS) strategy at Beijing Genomic Institute (BGI, Shenzhen, China). The filtered reads were assembled, scaffolded, gap filled, and validated using SOAP*denovo* version 2.04 (BGI, Shenzhen, China), SSPACE version 2.0 (BaseClear, Leiden, The Netherlands), Gap-Filler version 1.10 (BaseClear, Leiden, The Netherlands), and BWA version 0.7.4 [31], respectively. The NCBI Prokaryotic Genomes Automatic Annotation Pipeline [32] was used for gene annotation in preparation for data submission to GenBank (http://www.ncbi.nlm.nih.gov/genomes/static/Pipeline.html).

### 3.6. Analytical Methods

The HPLC analysis of DBP and PA was performed as described previously [11].

### 3.7. Nucleotide Sequence Accession Number

The whole-genome shotgun project has been deposited at GenBank under the accession number JPYZ00000000, and it is the first version described in this paper.

## 4. Conclusions

In conclusion, a strain (QH-12) capable of degrading DBP was isolated from activated sludge. 16S rRNA gene sequence analysis identified the QH-12 as belonging to the genus *Gordonia*. The experimental results showed that DBP could be rapidly degraded by *Gordonia* sp. QH-12 and the biodegradation kinetics could be described well using a modified Gompertz model. It was proposed that the metabolism of DBP by *Gordonia* sp. strain QH-12 occurred via the formation of phthalic acid (PA), but only a little amount of PA was detected. These results suggested that a novel degradation pathway was present in the strain. The QH-12 genome analysis revealed the underlying mechanism and degradation pathways for PAEs. Information about the genome sequence of *Gordonia* sp. QH-12 offered an opportunity to understand the diversity of *Gordonia* and the mechanism of PAEs degradation of this important genus. Further experiments are needed to verify annotated functional roles of degradation genes as well as the expansion of the detailed analysis of the genomes from genus *Gordonia*.

## Figures and Tables

**Table 1:** Comparison of calculated the lag phase, maximum transformation rate and the correlation coefficient using the modified Gompertz model at different initial DBP concentrations.

Initial Concentration/(mg/L)	*λ*/h	*R*m/(mg/L/h)	*R*^2^
100	3.825	8.673	0.980
200	5.205	18.085	0.989
300	7.616	29.449	0.998
500	10.974	50.212	0.990
750	11.428	66.367	0.994

**Table 2:** Substrate utilization profile for strain QH-12.

Substrate	Utilization	Substrate	Utilization	Substrate	Utilization
DMP	+	DOP	++	MBP	++
DEP	++	DIOP	+	Phthalic acid	−
DBP	++	DEHP	+	Protocatechuic acid	−

(++) vigorous growth; (+) growth; (−) no growth. The concentration of each substrate was 200 mg/L.
